# Three-year outcomes for women newly initiated on lifelong antiretroviral therapy during pregnancy – Malawi option B+

**DOI:** 10.1186/s12981-023-00523-1

**Published:** 2023-06-12

**Authors:** Maganizo B. Chagomerana, Bryna J. Harrington, Bethany L. DiPrete, Shaphil Wallie, Madalitso Maliwichi, Austin Wesevich, Jacob N. Phulusa, Wiza Kumwenda, Allan Jumbe, Mina C. Hosseinipour

**Affiliations:** 1 UNC Project-Malawi, Private Bag A-104, Lilongwe, Malawi; 2 Department of Medicine, University of North Carolina at Chapel Hill, Chapel Hill, NC USA; 3 Johns Hopkins Dept. of Gynecology & Obstetrics, Baltimore, MD USA; 4 Department of Epidemiology, Gillings School of Global Public Health, University of North Carolina at Chapel Hill, Chapel Hill, NC USA; 5 Kamuzu University of Health Sciences, Blantyre, Malawi; 6 Section of Hematology/Oncology, University of Chicago, Chicago, IL USA

**Keywords:** Pregnancy, HIV, Malawi, Option B+, Vertical transmission, Antiretroviral therapy, Birth outcomes

## Abstract

**Introduction:**

Antiretroviral therapy (ART) is very effective in preventing vertical transmission of HIV but some women on ART experience different virologic, immunologic, and safety profiles. While most pregnant women are closely monitored for short-term effects of ART during pregnancy, few women receive similar attention beyond pregnancy. We aimed to assess retention in care and clinical and laboratory-confirmed outcomes over 3 years after starting ART under Malawi’s Option B + program.

**Methods:**

We conducted a prospective cohort study of pregnant women newly diagnosed with HIV who started tenofovir disoproxil fumarate/emtricitabine/efavirenz (TDF/3TC/EFV) for the first time at Bwaila Hospital in Lilongwe, Malawi between May 2015 and June 2016. Participants were followed for 3 years. We summarized demographic characteristics, pregnancy outcomes, and clinical and laboratory adverse events findings using proportions. Log-binomial regression models were used to estimate the overall risk ratios (RR) and the corresponding 95% confidence interval (CI) for the association between index pregnancy (i.e. index pregnancy vs. subsequent pregnancy) and preterm birth, and index pregnancy and low birthweight.

**Results:**

Of the 299 pregnant women who were enrolled in the study, 255 (85.3%) were retained in care. There were 340 total pregnancies with known outcomes during the 36-month study period, 280 index pregnancies, and 60 subsequent pregnancies. The risks of delivering preterm (9.5% for index pregnancy and13.5% for subsequent pregnancy: RR = 0.70; 95% CI: 0.32–1.54), or low birth weight infant (9.8% for index pregnancy and 4.2% for subsequent pregnancy: RR = 2.36; 95% CI: 0.58–9.66) were similar between index and subsequent pregnancies. Perinatally acquired HIV was diagnosed in 6 (2.3%) infants from index pregnancies and none from subsequent pregnancies. A total of 50 (16.7%) women had at least one new clinical adverse event and 109 (36.5%) women had at least one incident abnormal laboratory finding. Twenty-two (7.3%) women switched to second line ART: of these 64.7% (8/17) had suppressed viral load and 54.9% (6/17) had undetectable viral load at 36 months.

**Conclusion:**

Most of the women who started TDF/3TC/EFV were retained in care and few infants were diagnosed with perinatally acquired HIV. Despite switching, women who switched to second line therapy continued to have higher viral loads suggesting that additional factors beyond TDF/3TC/EFV failure may have contributed to the switch. Ongoing support during the postpartum period is necessary to ensure retention in care and prevention of vertical transmission.

## Introduction

Option B+, a Prevention of Mother to Child Transmission (PMTCT) of HIV program, has ensured timely access to antiretroviral therapy (ART) among pregnant and breastfeeding women living with HIV in numerous countries. Under Option B+, pregnant and breastfeeding women living with HIV are offered lifelong ART regardless of their CD4 cell count or WHO clinical stage [1]. The primary goal of Option B + is PMTCT for both the current as well as subsequent pregnancies, which may eventually lead to elimination of mother to child transmission (EMTCT).

In 2019, 95% of the estimated pregnant women living with HIV in eastern and southern Africa, regions with high burdens of HIV, received ART [2]. This increased ART uptake among pregnant and breastfeeding women in eastern and southern Africa has greatly reduced the rate of vertical HIV transmission from 18% to 2010 to 8% in 2019 [2]. Some countries with a low burden of HIV have already met the validation criteria for EMTCT [3]. In addition to preventing vertical transmission, ART reduces maternal morbidity and mortality, and improves birth outcomes [4, 5]. These benefits are more evident when pregnant and breastfeeding women living with HIV are initiated on a potent, safe, and well-tolerated ART regimens early in pregnancy or as soon as they are diagnosed while pregnant or breastfeeding [6–8].

Malawi started implementing Option B + in 2011 with a once-daily fixed-dose combination of tenofovir, lamivudine and efavirenz (TDF/3TC/EFV) as the first-line ART regimen. TDF/3TC/EFV is efficacious, safe, and well-tolerated in the general adult population and has been recommended by the World Health Organization (WHO) [9, 10] However, due to physiological and humoral changes associated with pregnancy, women may experience a different virologic, immunologic, and safety profile with antiretroviral use, requiring monitoring for both short- and long-term effects of TDF/3TC/EFV on pregnant and breastfeeding women, fetuses, and children. In short-term evaluations of women on Option B + in Malawi at delivery, 6 months, and 12 months after initiating ART, 69–90% had suppressed viral load (VL) (VL < 1000 copies/ mL); 19% experienced at least one laboratory-confirmed adverse event over 12 months after starting ART; and 35% of women failing on TDF/3TC/EFV experienced HIV resistance primarily to the nonnucleoside reverse transcriptase inhibitor class of drugs over 6 months after starting ART [11–13]. During the first 12 months, few pregnant and breastfeeding women on TDF/3TC/EFV developed anemia (8%) and hepatotoxicity (8%) but none had elevated creatinine indicative of renal insufficiency [13].

Although data demonstrate few adverse events and increasing proportions of viral suppression 12 months after starting ART under Option B + in Malawi, data beyond 12 months are scant. Similarly, birth outcomes for subsequent pregnancies for mothers who started ART on Option B + in Malawi during pregnancy have not been described when identified from conception in a prospective manner. In this analysis, we used data from a cohort of pregnant women newly diagnosed with HIV and who initiated TDF/3TC/EFV under Option B + in Malawi to assess clinical and laboratory confirmed adverse events, CD4 count, and VL suppression over 3 years after starting ART. We also compared birth outcomes between index and subsequent pregnancies.

## Methods

### Study design and population

This analysis used data from the “Safety, Suppression, Second-line, Survival” (S4) study; NCT02249962. S4 was a prospective observational cohort study of pregnant women living with HIV aged ≥ 16 years old (includes adults and emancipated minors) engaging in antenatal care at Bwaila Hospital, in Lilongwe, Malawi from May 2015 to June 2019. Bwaila Hospital is a high-volume hospital delivering over 12,000 babies annually. This analysis used data from a sub-cohort of newly diagnosed pregnant women initiating TDF/3TC/EFV.

The S4 study was approved by Malawi National Health Sciences Research Committee and the University of North Carolina at Chapel Hill institutional review board. All women enrolled in the study provided informed consent.

### Study procedures

#### Recruitment and baseline activities

The recruitment of this sub-cohort of pregnant women newly diagnosed with HIV took place in the first year of the study (May 2015–June 2016) and followed all women antenatally and mother-child pairs postnatally for a total of 36 months from enrollment.

Following the Malawi Ministry of Health integrated PMTCT/ART guidelines, all pregnant women attending their first antenatal care (ANC) visit for the current pregnancy received HIV testing using the opt-out approach [14, 15]. Women who tested positive for HIV underwent ART education and counseling and were started on TDF/3TC/EFV on the same day. Women who consented to participate in the study provided blood samples for complete blood count (including hemoglobin, platelet count, absolute lymphocyte count, absolute neutrophil count, and total white blood cell count), blood chemistries (including creatinine, phosphate, alanine aminotransferase (ALT), and total bilirubin), CD4 cell count, HIV VL, and storage for HIV genotyping. The study personnel also administered a questionnaire to the participants to collect social and demographic information.

#### Follow up schedule and activities

All women enrolled in the study were asked to report at the hospital for scheduled visits as follows: monthly for the first 6 months, once every 3 months from 6 months to 12 months, and then once every 6 months until 36 months were completed. Clinical activities during these scheduled visits included physical examination, ART refill, and the following assessments: ART adherence by self-report confirmed with pill count, clinical adverse events, and laboratory toxicities. During the scheduled visits, blood samples for complete blood count and chemistries were collected once every 3 months for the first 6 months, and every 6 months thereafter. Blood samples for HIV VL testing were collected every 6 months during the first year, at delivery, and annually thereafter. CD4 cell count was evaluated annually. Safety laboratory assays including CD4 cell count and HIV VL were conducted at the UNC Project clinical research laboratory. CD4 cell count and HIV VL testing were conducted using Becton Dickinson FACScounts and the Abbott M2000, respectively. For all scheduled visits, participants were counselled on ART adherence, received additional care as part of research procedures from our staff in a less crowded clinic compared to government clinic, and received money for transport reimbursement. Women who missed visits were called or visited in the community to return for care. We considered a woman as lost to follow up if they could not be reached by telephone or be located in the community on three tracing attempts and did not report to clinic within 3 months of their scheduled date. For this analysis, our 36 month retention in care was defined as being alive and on ART to align with Malawi Ministry of Health guidelines [16].

### Outcomes measurement and definitions

We classified birth outcomes as live born infants and non-live born infants. The non-live infants included miscarriages (spontaneous and induced < 20 weeks gestation) and stillbirths (≥ 20 weeks gestation). Gestational age was calculated based on last menstrual period (LMP) because few women had both LMP and ultrasound dating completed. Additional birth-related outcomes of interest included preterm birth (< 37 weeks gestation), low birth weight (< 2500 g), neonatal death (died within 28 days of delivery), congenital anomalies, perinatally acquired HIV (tested and confirmed HIV positive ≤ 6 weeks), and safety of TDF/3TC/EFV. HIV status for children was assessed at birth, 6 weeks, 6 months, 12 months, and 24 months using dried blood sample PCR or rapid HIV-1/2 antibody testing, depending on age as per national testing guidelines for exposed children [15].

On safety outcomes, we focused on adverse events associated with the medical treatment (TDF/3TC/EFV). Adverse events were classified as clinical or laboratory. Clinical adverse events (unintended signs, symptoms, or diseases) included severe (grade 3), life-threatening (grade 4), or death (grade 5). Laboratory adverse events indicative of treatment toxicity were graded using the National Institute of Allergy and Infectious Diseases Division of AIDS (DAIDS) Table for Grading the Severity of Adult and Pediatric Adverse Events [17]. The laboratory adverse events included blood chemistries and hematologic values of grade 2 or higher. In addition to these graded events, we also included new obstetrical or postpartum medical complications and development of new WHO stage 3 or 4 conditions. In this analysis, we focused on incident adverse events defined as the first occurrence of a particular condition in each individual after enrollment except for obstetric events. We did not treat subsequent events as incidents due to the difficulty in differentiating these events from persistent conditions.

### Statistical analysis

Descriptive statistics were used to summarize the distribution of baseline socio-demographic and clinical characteristics of the women. We calculated the incidence of subsequent pregnancies and the corresponding 95% confidence interval (CI). The cumulative follow up time for incidence of subsequent pregnancies was calculated from index pregnancy delivery date to the last known date of follow up or study end date. Log-binomial regression models were used to estimate the overall risk ratios (RR) and the corresponding 95% CI for the association between index pregnancy (i.e. index pregnancy vs. subsequent pregnancy) and preterm birth, and index pregnancy and low birthweight.

All adverse birth outcomes and adverse infant outcomes were calculated as a proportion of total births delivered and total number of live born infants, respectively. We calculated the number of new clinical adverse events and the proportion of women who developed at least one adverse clinical event over the follow-up period. Similar calculations were done for laboratory adverse events.

Medians and interquartile ranges (IQR) were used to summarize the distribution of CD4 cell count over the study period. We calculated the proportion of women who switched from first line therapy (TDF/3TC/EFV) to second line therapy. We also calculated the proportion of women with suppressed VL (VL < 1000 copies/mL) and undetectable VL (VL < 40 copies/mL) over the study period.

All analyses were conducted in SAS version 9.4 (SAS Institute, Cary, NC, USA).

## Results

### Characteristics of the study population

A consort diagram of participant recruitment and engagement in the S4 study has been described elsewhere [13]. Over one year, 299 pregnant women newly diagnosed with HIV were enrolled in the study and initiated on standard first line ART (TDF/3TC/EFV). The median age at enrollment was 27 years (IQR 23–30) and median gestational age at first presentation was 22.1 weeks (IQR: 17.9–26.9). Most of the women enrolled were in relatively good health (WHO disease stage 1) (94.3%), married or living with a partner (89.0%), and had been pregnant before (81.9%) (Table 1).

**Table 1:** Social-demographic and baseline clinical characteristics of women enrolled in S4 study

	Number of Women enrolled (N = 299)	
***Social-demographic characteristics***		
Age in years (median (IQR))	27	(23–30)
Gestational age at 1st clinic visit in weeks (median [IQR])	22.1	(17.9–26.9)
	N	(%)
Marital status		
Single	10	(3.3)
Married or living with partner	266	(89.0)
Separated /divorced /widowed	23	(7.7)
Education		
No Schooling	9	(3.0)
Primary	164	(54.9)
Secondary	117	(39.1)
Tertiary	9	(3.0)
Distance to clinic		
< 1 km	131	(43.8)
≥ 1 km	168	(56.2)
WHO clinical stage		
1	282	(94.3)
2	13	(4.4)
3	4	(1.3)
Parity		
Nulliparous	54	(18.1)
Primiparous	105	(35.1)
Multiparous	140	(46.8)
***Relationship with sexual partner for current pregnancy***		
Still in relationship		
Yes	287	(96.0)
No	12	(4.0)
Is primary sex partner		
Yes	293	(98.0)
No	6	(2.0)
Live with partner		
Yes	262	(87.6)
No	37	(12.4)
*Sexual partner HIV status*		
Positive	42	(14.0)
Negative	69	(23.1)
Missing/Unknown	188	(68.9)

Forty-four (14.7%) participants did not complete the required 36 months of follow-up because they were lost to follow up (15/44, 34.1%), relocated from the study catchment area (14/44, 31.8%), withdrew consent (8/44, 18.2%), or died (7/44, 15.9%).

### Pregnancy outcomes

As previously reported, of the 299 pregnant women enrolled, 19 (6.4%) had missing birth outcomes for the index pregnancy (5 lost to follow-up, 3 withdrew consent, and 11 relocated) [13]. There were 340 total pregnancies during the 36-month study period, and 345 infants were born (5 were from twin gestations). Of the 345 infants, 284 were from the 280 index pregnancies, and 61 infants from 60 subsequent pregnancies.

#### Index pregnancies

Among the 284 infants who were delivered from 280 index pregnancies, 19 (6.3%) were non-live births (14 stillbirths and 5 spontaneous miscarriages). Most live born index infants were spontaneous vaginal deliveries (222/265, 83.8%). Six index infants (2.3%) were diagnosed with perinatally acquired HIV (Table 2). Of the 265 live births, 21 (7.9%) infants had missing birth weight and 1 (0.4%) infant had missing gestational age. Among the live births, the risk of preterm birth and low birth weight for index pregnancies were 0.09 (25/264); 95% CI: 0.06–0.13 and 0.10 (24/244); 95% CI: 0.06–0.14, respectively.

**Table 2:** Summary of live birth infants for index and subsequent pregnancies

	Index pregnancy N = 265		Subsequent pregnancy N = 54	
Sex				
Male	138	(52.3)	21	(38.9)
Female	126	(47.7)	33	(61.1)
Missing	1		0	
Delivery method				
Spontaneous vaginal	222	(83.8)	44	(81.5)
Vaginal forceps	1	(0.4)	0	
Vaginal vacuum	3	(1.1)	0	
Planned Cesarean section	8	(3.0)	3	(5.5)
Cesarean section after labor	30	(11.3)	7	(13.0)
Unknown	1	(0.4)	0	
Multiple gestation	7	(2.6)	2	(3.7)
Gestation age at delivery				
Term ( > = 37 weeks)	239	(90.5)	45	(86.5)
Preterm (< 37 weeks)	25	(9.5)	7	(13.5)
Missing gestation age	1		2	
Birthweight				
Normal (≥ 2500 g)	220	(90.2)	46	(95.8)
Low (< 2500 g)	24	(9.8)	2	(4.2)
Missing birthweight	21		6	
Congenital anomalies^*^	6	(2.3)	1	(1.9)
Neonatal deaths	5	(1.9)	1	(1.9)
Perinatally acquired HIV^†^	6	(2.3)	0	(0)

^*^ 2 musculoskeletal/extremities and 1 for each of the following: extra digits on both hands, hydrocephalus, tongue tie, and weakness of left limb

^†^ Tested and confirmed HIV positive ≤ 6 weeks after delivery

#### Subsequent pregnancies

During the study follow-up period, 56 women (18.7%) had at least one repeat pregnancy (total repeat pregnancies = 60) delivering 61 infants. From index delivery to the end of the study, the incidence of subsequent pregnancy was 7.63 (95% CI: 5.91–9.85) pregnancies per 100 person-years. Of the 61 subsequent infants, 7 (11.5%) were non-live births (5 miscarriages and 2 stillbirths). Among the subsequent live born infants, 44 (81.5%) were spontaneous vaginal deliveries. No perinatally acquired HIV was detected among subsequent pregnancies (Table 2). Six (11.1%) live birth infants had missing birth weight and 2 (3.7%) infants had missing gestational age. Among live births, the risk of preterm birth and low birth weight for subsequent pregnancies was 0.13 (7/52); 95% CI: 0.04–0.23 and 0.04 (2/48); 95% CI: 0.00–0.10, respectively.

Index and subsequent pregnancies had similar risk of delivering a non-live born (RR = 0.58; 95% CI: 0.26–1.33) or preterm (RR = 0.77; 95% CI: 0.41–1.46) infant. Among live births, the risks of delivering preterm (RR = 0.70; 95% CI: 0.32–1.54), or low birth weight infant (RR = 2.36; 95% CI: 0.58–9.66) were also similar between index and subsequent pregnancies.

### New clinical and laboratory adverse events

Over the 36 months of the study, 82 new clinical adverse events of grade ≥ 3 were observed in 50 (16.7%) women. These clinical adverse events included 53 grade 3 and 4, 22 obstetrical/post-partum complications, and 7 deaths. The majority of grade 3 and 4 clinical events were infectious in etiology [Malaria (9 cases), pneumonia (7 cases), Sepsis (8 cases), tuberculosis (4 cases), Urinary tract infection (2 cases), trichomoniasis (2 cases), acute gastroenteritis (2 cases), tonsillitis (1 case), and pelvic inflammatory disease (1 case)]. With respect to obstetrical/post-partum complications, we identified the following: spontaneous abortion (11 cases), puerperal sepsis (4 cases), pre-eclampsia (2 cases), retained product of conception (2 cases), abruptio placenta (1 case), infected caesarian section wound (1 case), and ruptured ectopic pregnancy (1 case). Of the 82 new clinical events, 9 (11%) were related to ART treatment resulting in three women permanently discontinuing or changing therapy. Additionally, 149 new laboratory adverse events indicative of toxicity were observed in 109 (36.5%) women. The most common laboratory toxicities were low hemoglobin (38.9%, n = 58) and elevated ALT (24.8%, n = 37) (Table 3).

**Table 3:** New clinical and laboratory adverse events observed over 3 years

	Number of adverse events N (%)	
**Clinical adverse events grade ≥ 3**	**82** ^*^	
Grade 3: severe	45	(54.9)
Grade 4: life threatening	8	(9.8)
Grade 5: death^†^	7	(8.5)
Obstetrical/postpartum medical condition	22	(26.8)
**Laboratory event grade ≥ 2**	**149**ǂ	
Low hemoglobin	58	(38.9)
Elevated alanine aminotransferase (ALT)	37	(24.8)
Low absolute neutrophil count	18	(12.1)
Low platelets	13	(8.7)
Elevated bilirubin (total)	10	(6.7)
Low Phosphate	6	(4.0)
Low white Blood Cells (WBC)	4	(2.7)
Low absolute lymphocyte count	3	(2.0)
Elevated creatinine	0	(0.0)

^*^ 82 new clinical events of grade ≥ 3 were recorded in 50 women

^†^ Causes of death: 1 anemia, 1 jaundice, 1 miliary tuberculosis, 1 road accident, and 3 unknown

ǂ 149 new laboratory events of grade ≥ 3 were recorded in 109 women

### CD4 cell count and viral suppression

**Fig. 1:**
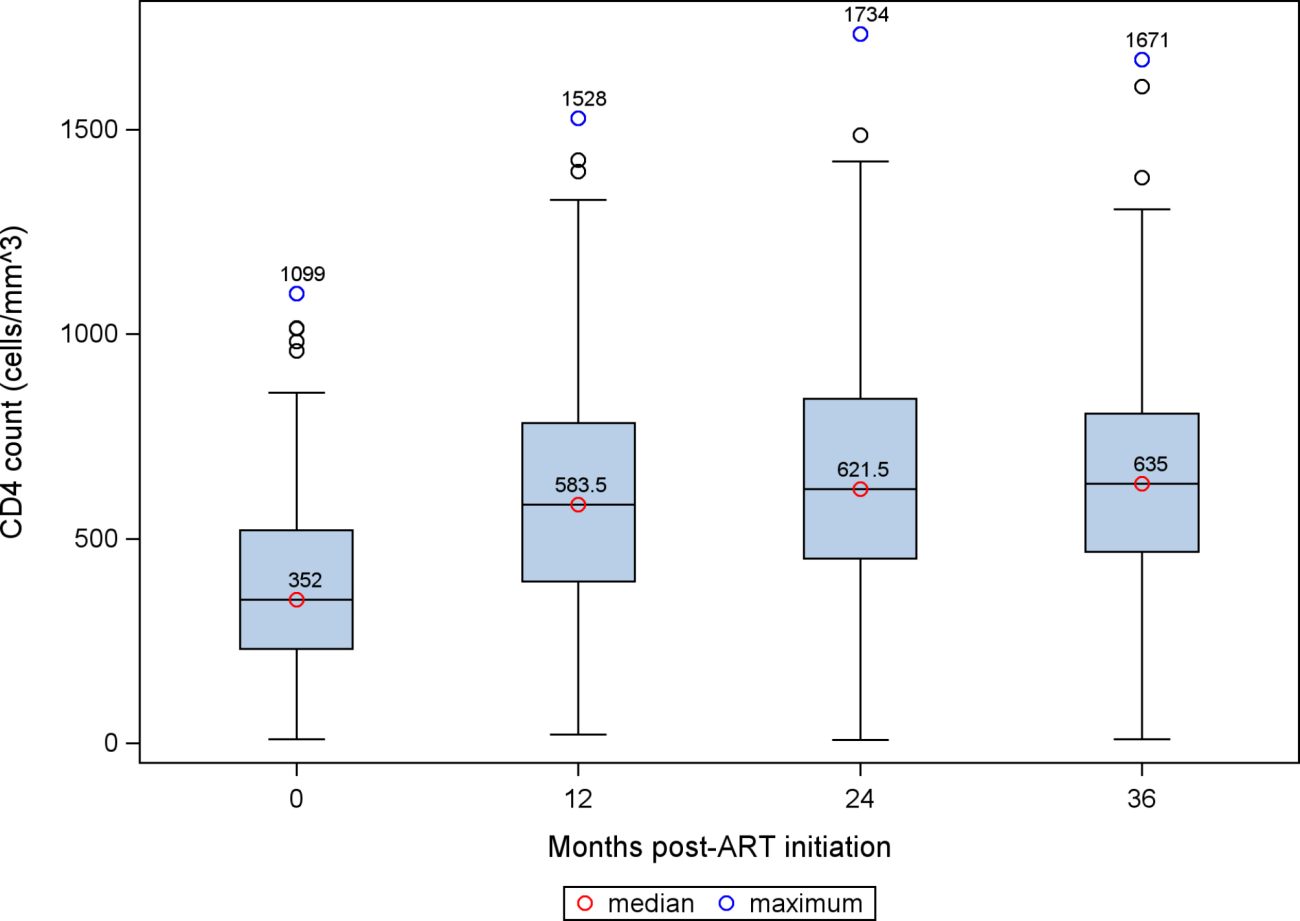
CD4 cell count distribution for women over the study period

**Fig. 2:**
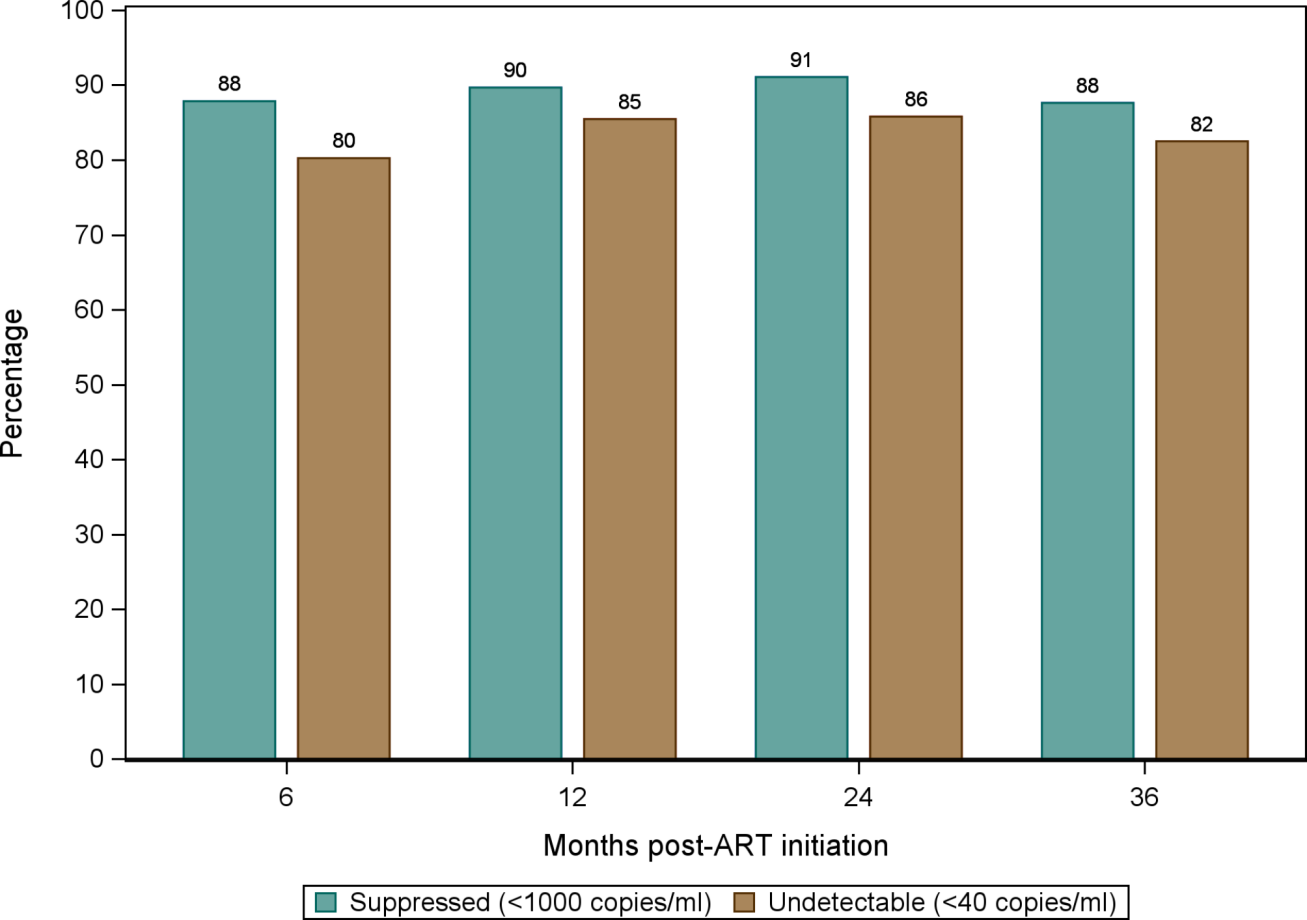
Proportion of women with suppressed viral load (VL < 1000 copies/mL) and undetectable viral load (VL < 40 copies/mL) over the study period

**Fig. 3:**
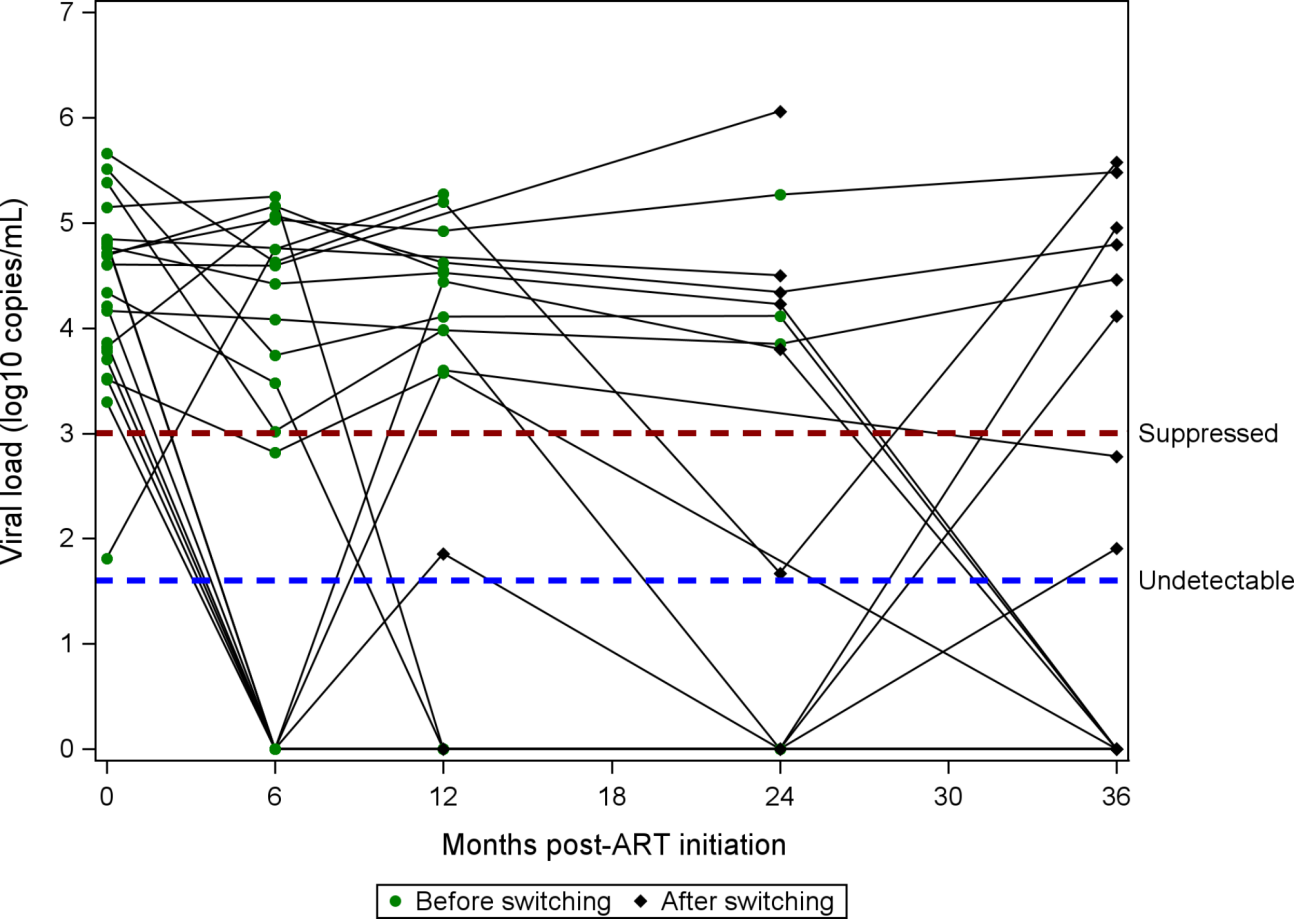
Viral load for women who switched to second line therapy

The median CD4 count at enrollment was 352 cells/mm^3^ (IQR: 231–550) and increased to 635 cells/mm^3^ (IQR: 469–843) at month 36 (Fig. 1). The proportion of women who had suppressed VL and undetectable VL at 36 months were 88% and 82%, respectively (Fig. 2). Of the 299 participants, 22 (7.3%) switched from first line therapy (TDF/3TC/EFV) to second-line therapy due to virologic failure (n = 10), clinician decision or participant request (n = 3), decreased or resolved toxicity (n = 1) and other reasons (n = 8). Virologic failures occurred between 8 and 25 months, with a median time to failure of 17.9 months (IQR: 9.8–21.9). Among the 22 women who switched to second line, 5 were lost to follow up and did not have subsequent VL at 36 months; 64.7% (8/17) had suppressed VL and 54.9% (6/17) had undetectable VL at 36 months (Fig. 3).

## Discussion

After 3 years of follow-up on a cohort of 299 pregnant women newly diagnosed with HIV and initiated on TDF/3TC/EFV, 284 infants were delivered from the index pregnancy and 61 from subsequent pregnancies. The risks of delivering non-livebirths, preterm, and low birth weight infants were similar between index and subsequent pregnancies. Few women experienced ART treatment-related clinical adverse events that required permanent discontinuing or changing of therapy. More than 85% of the women were retained in care through the study period and most of them had suppressed VL.

With widespread availability of safe and effective ART, more women living with HIV (WLHIV) are conceiving while on ART and giving birth to healthy and mostly HIV-negative infants. Some WLHIV are experiencing repeat pregnancies. In this study, over 15% of participants experienced at least one repeat pregnancy, comparable to rates of repeat pregnancies observed by the Surveillance of Antiretroviral Treatment in Pregnancy study in Italy (16.2% over 5 years), Women and Infants Transmission Study in North America (21.9% over 5 years), and the Kisumu Breastfeeding Study in Kenya (17.6% over 2 years) [18–20]. In Latin America and the Caribbean, 9.2% of WLHIV experienced repeat pregnancies between 2002 and 2011 [21]. While improved health among WLHIV has contributed to the rise in repeat pregnancies,[18, 21, 22] the desire for more children among WLHIV is also influenced by social factors such as age, parity, or pressure from partners, in-laws, and other relatives [20, 22, 23]. Thus, providing ART adherence and contraceptive counselling for WLHIV during and after they finish breastfeeding is critical for ensuring good maternal health and PMTCT in subsequent pregnancies.

Our finding that low hemoglobin was the most frequently occurring laboratory abnormality among pregnant women with HIV is not surprising. Anemia is highly prevalent among pregnant women in sub-Saharan Africa and pregnant women living with HIV are at higher risk of having anemia [24, 25]. In Malawi, approximately 40% of pregnant women have anemia [26]. Regardless of HIV status, anemia in pregnancy is associated with adverse birth outcomes such as maternal deaths, stillbirths, and low birth weight infants [27–29]. However, longer duration of ART use has been associated with reduced risk of developing anemia in clinical studies conducted in sub-Saharan countries including Malawi [30, 31]. In fact, none of the components in TDF/3TC/EFV are considered to lead to anemia based on available evidence [32, 33]. Low hemoglobin in some of the women on ART may have exacerbated the occurrence of adverse birth outcomes in this study.

Pregnant women living with HIV generally have elevated levels of liver enzymes compared to non-pregnant counterparts [34, 35]. Additionally, Efavirenz (EFV)–containing ART use among people living with HIV increases the risk of developing hepatotoxicity [36]. While 7.4% of women in the Promoting Maternal and Infant Survival Everywhere (PROMISE) trial had grade ≥ 2 elevated ALT after initiating EFV–containing ART,[37] 10% of pregnant women living with HIV on TDF/3TC/EFV ART regimen in our study developed new hepatotoxicity events of grade ≥ 2 over the 3 years of follow-up. Many of them were unsustained and did not require treatment suspension. However, the rates of hepatotoxicity have varied across studies due to differences in classification of hepatotoxicity and duration on ART. The risk of hepatoxicity persists with continued ART use, as the S4 study had 7% new hepatotoxicity events of grade ≥ 2 at 12 months but 10% at 36 months [13]. Similarly, studies in the United Kingdom and Ireland show increases in grade 1–4 hepatoxicity from 15% to 1 year to 30% at 5 years (and from 2.2 to 4.3%, respectively for grade 3–4 hepatotoxicity) [34].

Sustained viral suppression and strong immune systems among pregnant and breastfeeding women on ART are essential for preventing HIV transmission and good maternal health. In addition to the favorable side effect profile and fewer drug interactions associated with TDF/3TC/EFV,[38, 39] pregnant women in our study experienced rapid reductions in viral load and improvements in CD4 cell count. Most of the women in this study were virally suppressed within six months of ART initiation and the median CD4 cell count increased by over 200 cells/mm^3^ within a year of ART initiation. Among our participants who switched to second line treatment, viral suppression remained inadequate despite the change to an expected fully active regimen, likely suggesting adherence and other factors are responsible. To sustain these immunologic gains, pregnant and breastfeeding women should be counselled to adhere to treatment as discontinuation of treatment severely impairs immunologic parameters [11, 40].

The introduction of Option B + in Malawi dramatically increased the number of pregnant or breastfeeding women on ART after one year of implementation [41]. Despite this success, the program continues to experience poor retention in care, especially during the postpartum period [42, 43]. In a cohort of women who started ART under Option B + and never transferred to a different facility between Oct 1, 2011, and Dec 31, 2013, only 76.8%, 70.8%, and 69.7% were retained in care after 12 months, 24 months, and 36 months, respectively, representing a cumulative 30.3% loss to follow-up at 36 months [43]. Our finding of 85.3% retention in care at 36 months is therefore higher and may be indicative of improvement in retention over time as the Option B + program matured. However, the loss to follow up rate that we observed in our study may be an under estimate of the actual loss to follow up in the Option B + program. Participants in our study may have been motivated to remain in care by the transport reimbursement that was provided as part of participation, a less crowded clinic compared to government clinic, and additional care they received as part of research procedures from our staff. Nevertheless, our high retention rate provided an opportunity for more comprehensive evaluation of outcomes including virologic suppression and safety, than can be done in cohort studies that rely on routine data only.

While ART is now recommended for all pregnant and breastfeeding women, evidence regarding the relationship between ART and adverse pregnancy outcomes remains mixed [44, 45]. In this study, there were no differences in the risks of preterm birth and low birth weight between women who started TDF/FTC/EFV during pregnancy (index pregnancy) and women who were on TDF/FTC/EFV during conception (subsequent pregnancies). When compared with other 3-drug ART (generally zidovudine/lamivudine/nevirapine (ZDV/3TC/NVP) at conception in Botswana, women on TDF/FTC/EFV at conception had fewer stillbirths (4.9% vs. 6.4%), and fewer small for gestation age infants (8% vs. 24%) but similar rates of preterm births (28% vs. 32%) and very preterm births (10% vs. 12%), although these differences were not statistically significant in multivariable analysis [46]. In South Africa, both preconception TDF/(3TC/FTC)/EFV and post-conception TDF/(3TC/FTC)/EFV were not associated with preterm births compared with nevirapine-based regimen or other 3-drug EFV based regimens (abacavir(ABC)/3TC/EFV, ZDV/3TC/EFV), and stavudine(d4T)/3TC/EFV)) [47]. Our findings of no difference in the risks of preterm birth and low birth weight between index and subsequent pregnancies conflict with those from a meta-analysis of 11 studies and PROMISE 1077BF/1077FF trials [48, 49]. The meta-analysis of 11 studies demonstrated a significantly increased risk of preterm delivery (< 37weeks) and low birth weight (< 2500 g) when conceiving on ART compared to initiating ART during pregnancy [48]. Among women enrolled in the PROMISE 1077BF/1077FF trials, women who conceived while on ART had more than twice the risk of delivering infants with low birth weight compared to those who were initiated on ART during pregnancy [49]. This conflicting evidence on the risk of adverse pregnancy outcomes can be attributed to heterogeneities across studies in design, population, timing of ART initiation, and ART regimens. Of note, our finding of no differences in birth outcomes between index and subsequent pregnancies may be due to lack of statistical power. This study was not powered to detect differences in birth outcomes. In addition, the number of pregnancy losses that we observed for the index pregnancy may be an underestimate because women were enrolled at any gestation age during the index pregnancy. As a result, the number of pregnancy losses for index pregnancies did not include early pregnancy losses as was included for subsequent pregnancies.

Most of the women who started a once-daily fixed-dose combination of TDF/3TC/EFV during pregnancy in the Option B + program in Malawi were retained in care through the three years of our study. Some of the women had subsequent pregnancies with similar birth and infant outcomes as index pregnancies, which suggests that TDF/3TC/EFV is safe among pregnant women regardless of the timing of initiation i.e., before conception vs. during pregnancy. Although some women experienced clinical and laboratory adverse events, most of the frequently observed adverse events such as low hemoglobin are common among pregnant women and cannot be fully attributed to TDF/3TC/EFV. Few women experienced virologic failure, and this typically occurred during the first year on ART. Ongoing support for women in the postpartum period should be encouraged to ensure retention in care and prevention of vertical transmission.

## Data Availability

The datasets used during the current study are available from the corresponding author on reasonable request.

## Abbreviations

ABC: Abacavir
ALT: Alanine aminotransferase
ART: Antiretroviral therapy
CI: Confidence interval
d4T: Stavudine
DAIDS: National Institute of Allergy and Infectious Diseases Division of AIDS
EMTCT: Elimination of mother to child transmission
IQR: Interquartile range
PMTCT: Prevention of mother to child transmission
PROMISE: Promoting Maternal and Infant Survival Everywhere Study
RR: Risk ratio
S4: Safety, suppression, second-line, survival
TDF/3TC/EFV: Tenofovir disoproxil fumarate/emtricitabine/efavirenz
VL: Viral load
WHO: World Health Organization
WLHIV: Women living with HIV
ZDV/3TC/NVP: Zidovudine/lamivudine/nevirapine
